# Pawnobiome: manipulation of the hologenome within one host generation and beyond

**DOI:** 10.3389/fmicb.2015.00697

**Published:** 2015-08-04

**Authors:** Jameson D. Voss, Juan C. Leon, Nikhil V. Dhurandhar, Frank T. Robb

**Affiliations:** ^1^United States Air Force School of Aerospace Medicine, Epidemiology Consult Service, Wright Patterson AFBOH, USA; ^2^Department of Nutritional Sciences, Texas Tech UniversityLubbock, TX, USA; ^3^Department of Microbiology and Immunology, University of MarylandBaltimore, MD, USA

**Keywords:** evolution, microbiome, hologenome, microbiota, pawnobe

## Metaorganisms and hologenome theory of evolution

A metaorganism is a collection of interacting organisms where the sum is not the same as the simple addition of the individual isolated parts (Relman, 2008; Webster, 2014). In fact, the gut, nasal, and lung microbiome all influence human phenotype (Redinbo, 2014). More specifically, the human gut microbiome has been linked to brain activity and to behavior (Collins et al., 2012). Similarly, *Bacillus amyloliquefaciens* supplementation improves feed conversion in chickens comparable to antibiotic growth promoters, by increasing villus height and crypt depth throughout the small intestine (Lei et al., 2015). It is apparent that phenotypic changes in the metaorganism influence the entire commensal unit.

The hologenome theory of evolution (HTE) asserts that a unit of selection is the holobiont which includes both the host and all its associated microbiota combined (Zilber-Rosenberg and Rosenberg, 2008). Some established components of the microbiota form a stable connection with the host; so, the entire holobiont is selected simultaneously with each passing host generation. The evolutionary fate of the holobiont unit is further linked with reliable vertical transmission of the microbiota whenever the host produces offspring.

The HTE is an important step forward in considering the evolutionary relevance of the wild-type microbiota, but it is not meant to characterize opportunities in deliberately manipulating and selecting microbes. Additionally, some microbes do not fit well within the HTE because they do not reliably transmit vertically, or they only influence host phenotype transiently. For instance, in one study, a yogurt probiotic altered bacterial carbohydrate metabolism markers without altering the species composition of the fecal bacteria (McNulty et al., 2011; Sanders et al., 2013). Similarly, fecal transplant appears promising for diabetes treatment, but thus far, has only been shown to improve insulin sensitivity temporarily (Vrieze et al., 2013). While a majority of the gut microbes in humans are stable day to day (Lozupone et al., 2012), only 60% of strains are durable beyond 5 years (Faith et al., 2013). These observations are the basis for our concept of the “Pawnobiome,” defined as the subset of the microbiome that is purposefully managed for manipulation of the host phenotype, which includes individual microbes named “pawnobes.”

## Characteristics of the pawnobiome

As we are defining it, the pawnobiome exists at a border between a stable relationship with the host and an unstable one. If a microbe is in a stable symbiosis which cannot be manipulated independently from the host, it is not a part of the pawnobiome. In other words, the pawnobiome is at the critical interface between temporary and permanent residence in the hologenome. By allowing both phenotype conservation and innovation, this criticality is likely an important factor determining evolvability of the hologenome (Torres-Sosa et al., 2012). Unlike the microbiota in the HTE, the pawnobiome is not strictly dependent upon a particular host's survival or generation time and can evolve independently and more rapidly than the host. The pawnobiome theory of evolution (PTE) is that as artificial evolution occurs within the pawnobiome, the host phenotype can be substantially altered within a single host generation.

Further, the pawnobes are also genetically adaptable. Gut bacteria, for instance, are known to be hypermutable *in vitro* (LeClerc et al., 1996; Lee et al., 2008) and an experimental *Escherichia coli* model progressed through potentiation, actualization, and refinement over 33,000 generations to gain a novel metabolic function (Blount et al., 2012). With *in vivo* observations, gut bacteria have shown substantial adaptability over a wide range of timescales (Quercia et al., 2014). In addition to criticality and modifiability, a third important feature of the pawnobes is transmissibility. For instance, if a pawnobe enhanced fitness of the host, widespread horizontal transmission could be possible [exemplified by stool donor banks (Burns et al., 2015)].

The term “pawn” has many connotations, but characteristics of criticality, transmissibility and adaptability are particularly relevant to the present theory. The term “pawn” reflects exchange of goods with lending and trade. The analogy can be extended to pawn shops, which exist at the border between regulated commercial exchange and unregulated barter. Further, a broker can reappraise, repackage, and combine with other goods to alter the value based on observable characteristics and transmit to a new owner (i.e., host). Similarly, these features of criticality, transmissibility, and modifiability are also seen in the game of chess. Here, pawns are the strategically important, least glorious pieces that make up the front line, buffering the more valuable pieces that one can control with the pieces one cannot (i.e., criticality). Pawns are captured in a gambit (i.e., transmissibility). Finally, if they survive and advance to the end of the board, they can be upgraded (i.e., modified) to a more functional piece during a single game.

Ultimately, the prefix “pawn” carries the connotation of strategic domestication. The term “domesticated microbiome,” is similar with “pawnobiome,” but we argue it is less precise because it suggests every microbe interacting with the host is domesticated, which would be unusual at the present time since undomesticated microbiota are still predominant.

## Opportunities in pawnobe selection

In 1859, Darwin and Bynum devoted the first chapter of “On the Origin of Species” to the variation in domesticated plants and animals. The concluding remarks of chapter 1 are a remarkable foresight into the current groundbreaking developments that are revealing the impact of artificial selection of commensal microbial species. He wrote, “… the most important point of all, is, that the animal or plant should be so highly useful to man, or so much valued by him, that the closest attention should be paid to even the slightest deviation in… each individual.”

Darwin described these insights on domesticated species separately from his observations on wildlife; similarly, the products of artificial selection in both kingdom Animalia (i.e., livestock) and Plantae (i.e., cultigens) have a separate name to describe their domestication as we are now also proposing for microbes (i.e., pawnobes).

Within a single host, the microbiome is a large (>100 fold more microbial genetic material than the human genome) and diverse population, (Ezenwa et al., 2012; Lozupone et al., 2012) which creates extraordinary opportunity for artificial selection. The pawnobiome population size can be amplified even more with a large number of hosts. For instance, some skin microbes appear to act as insect repellants (Ezenwa et al., 2012). After using the “closest attention” and selecting the most repellant microbes once, these microbes could be re-challenged and iteratively selected in a large number of hosts to repel medically important insect vectors.

The PTE proposes some elements of the microbiome are modifiable over a short time scale even if others are more difficult to change. Maximizing the utility in medicine, agriculture, and basic science will require new methods (e.g., trans-species artificial selection) to help optimize the pawnobiome.

The utility of the pawnobiome concept is experimentally testable. Because the transmissibility characteristic of pawnobes is not necessarily limited by species or other phylogenetic boundaries, a biocontained murine model could be used for multiple phenotypic traits that can be assessed in mice. Fortunately, mice are an ideal species to test artificial selection using fecal-oral transmission since they can be raised in sterile conditions, producing so-called gnotobiotic mice that are effective models for culturing the human gut microbiome (Goodman et al., 2011), and because they naturally engage in coprophagy (Ridaura et al., 2013). For instance, after administering a commercially successful chicken probiotic such as *B. amyloliquefaciens* (Lei et al., 2015) to gnotobiotic mice, frequent serial passage of the stool of mice with the highest feed conversion could continue until an optimized probiotic was isolated and sequenced.

Another application would create an optimized gut microbiome to resist the metabolic consequences of consumption of a high calorie diet by sedentary individuals. Already, observations in a human trial have identified so called, “super donors” who appear to provide a notably larger metabolic benefit to others upon stool transplant (Udayappan et al., 2014). We propose serial artificial selection could continue after identifying successful transplants. So, to begin the process, stool from a “super donor” could seed a large population of genetically homogenous mice eating a metabolically unhealthy diet (Ridaura et al., 2013). Then, after assessing target metabolic parameters (e.g., body weight, blood glucose, lipids, etc.) at frequent intervals, the stool from the leanest and otherwise healthiest mice could be redistributed to all other mice. Once metabolic parameters were optimized, the final product could be analyzed using community sequencing and metabolomics (Marcobal et al., 2013), and reference data from the Human Microbiome Project (Peterson et al., 2009). The entire stool, promising components, or individual products could proceed for further testing in animals and finally humans.

Aside from artificial selection, supplementation with probiotics (McNulty et al., 2011; Biagi et al., 2013; Sanders et al., 2013; Hulston et al., 2015), prebiotics (Biagi et al., 2013; Holscher et al., 2015), antibiotics (Cho et al., 2012), and dysbiotics (e.g., emulsifiers) (Chassaing et al., 2015) could alter host phenotype by changing the pawnobiome.

## Pawnobiome selection limitations and insights

While enhanced pawnobe selection holds promise, there are at least three limiting factors: observability, attribution, and permanence. Assessment of positive traits for selection requires detectable variation between otherwise genetically homogenous individuals. Additionally, the etiology of the observable variation needs to be attributable to the pawnobes that can be transmitted with the chosen method (i.e., fecal-oral). Another potential threat is permanence (i.e., undesirable chronic effects are not identifiable with short term selection of a transient phenotype). In humans, researchers use screening criteria to select donors without chronic disease for fecal transplant (Vrieze et al., 2013); such a technique could also be used to eliminate highly successful pathogens such as *Pseudomonas aeruginosa* (Markou and Apidianakis, 2014). Even if serial passage in mice led to the emergence of a pathogen, any short term desirable changes in phenotype could be evaluated to try to replicate the effect with a non-infectious vehicle.

Interestingly, the infectious disease risk in pawnobe artificial selection may be an under-recognized threat in other forms of artificial selection (e.g., antibiotic use, agricultural selection). While pawnobe selection occurs over smaller timescales, aggressive selection could pose the same risk for creating emerging pathogens (or releasing control of commensals that are conditional pathogens, like *Clostridium difficile*) over larger timescales. Furthermore, aggressive artificial selection among one species (e.g., a livestock species) could select microbiota that transmit traits by horizontal gene transfer (or other mechanisms) to multiple species simultaneously. Human gut bacteria are transferable between species (Sun et al., 2015), not surprising since humans share microbiota with their cohabiting dogs (Song et al., 2013; Udayappan et al., 2014).

Livestock breeds have undergone aggressive selection for metabolic characteristics that are commercially favorable. There is genetic evidence of selection for fat deposition in sheep (Moradi et al., 2012), feed efficiency in cattle (Bovine HapMap Consortium, 2009), and metabolic regulation in chickens (Rubin et al., 2010). Recently, a chicken breed that was originally commercialized in 1957 and another breed commercialized in 2005 were both raised simultaneously under the same management with the same food (Zuidhof et al., 2014). Under the same regime, the 2005 chicken breed weighed four times as much as the 1957 breed (Zuidhof et al., 2014). Such divergent phenotypes over a short period evidences aggressive recent selection of the host genome, and per the HTE, there should have been corresponding selection of the microbiota that influenced host phenotype in the same direction.

If there was a trans-species effect from aggressive selection in one species it might be detected in the body weight of interacting species. In fact, all animals with historical body weight records have gained weight in mid-life over the past several decades (Klimentidis et al., 2011). Several microbes associated with livestock are known to cause obesity in animals or are associated with human obesity. For instance, gut bacteria in the genus *Lachnospiraceae* are associated with cattle rumination and antibiotic weight gain in several species (Cho et al., 2012; Meehan and Beiko, 2014). Likewise, Adenoviruses (e.g., SMAM1, Ad-36), (Dhurandhar et al., 1992, 1997, 2001; Shang et al., 2014) *Toxoplasma gondii* (Carter, 2013; Reeves et al., 2013), and transmissible spongiform encephalopathies [i.e., Kuru, (Collinge et al., 2008) Creutzfeldt-Jakob Disease, (Manuelidis et al., 2009) Bovine Spongiform Encephalopathy (Strom et al., 2014), and scrapie agents (Kim et al., 1987)] are associated with obesity. Thus, shedding or acquisition of pawnobe species could provide new insights into infectious disease dynamics (Colizza and Vespignani, 2008) and other biological variation.

## Conclusions

In summary, the pawnobiome theory describes commensal microbiota that impact host phenotype but can be independently selected. Like other evolutionary developments in genetics and microbiota, pawnobe studies could be applied to agriculture (Thrall et al., 2011). Additionally, pawnobiome host interactions may provide insights for biological theories [e.g., autocenosis and democenosis (Savinov, 2011) symbiogenesis (Mereschkovsky, 1909; Kozo-Polyansky, 1924), synergistic selection (Corning and Szathmáry, 2015), teleonomy (Corning, 2014), endophyte studies (Taghavi et al., 2009), and the hygiene hypothesis (Strachan, 2000)]. Purposeful and cautious artificial selection could have broad ranging applications within biotechnology, health care, and evolutionary biology. Over time, new technologies and methods for strategic selection of the pawnobiome could accelerate this utility.

## Funding

The work was partially supported by the Air Force Office of Scientific Research by Grants AFOSR 03-S-28900 and 9550-10-1-0272 and by the NASA Exobiology Program (FTR).

### Conflict of interest statement

Jameson D. Voss, Juan C. Leon, and Frank T. Robb have nothing to declare. Nikhil V. Dhurandhar declares several patents in viral obesity and Adenovirus 36 including uses for E1A, E4orf1 gene and protein, and AKT1 inhibitor. He also declares ongoing grant support from Vital Health Interventions for determining anti-diabetic properties of E4orf1 protein.
